# Corrections

**Published:** 2004-09

In “Cause-Specific Mortality and the Extended Effects of Particulate Pollution and Temperature Exposure” by Goodman et al. [ Environ Health Perspect 112:179–184 (2004)], Figures 2–4 were incorrect; the corrected figures appear below. *EHP* regrets the errors.

The April 2004 news article “Reaching across the Border with the SBRP” [ Environ Health Perspect 112:A278–A279 (2004)] listed an incorrect URL for the University of Arizona website where visitors may download a Spanish-language environmental toxicology textbook. The correct URL is http://superfund.pharmacy.arizona.edu/outreach.html.

In the May 2004 toxicogenomics news article “Diet and DNA” [Environ Health Perspect 112:A404 (2004)], the European Nutrigenomics Organisation (NuGO) was described as “a network of 22 scientists” when in fact it is a network of 22 organizations.

*EHP* regrets the errors.

## Figures and Tables

**Figure 2:**
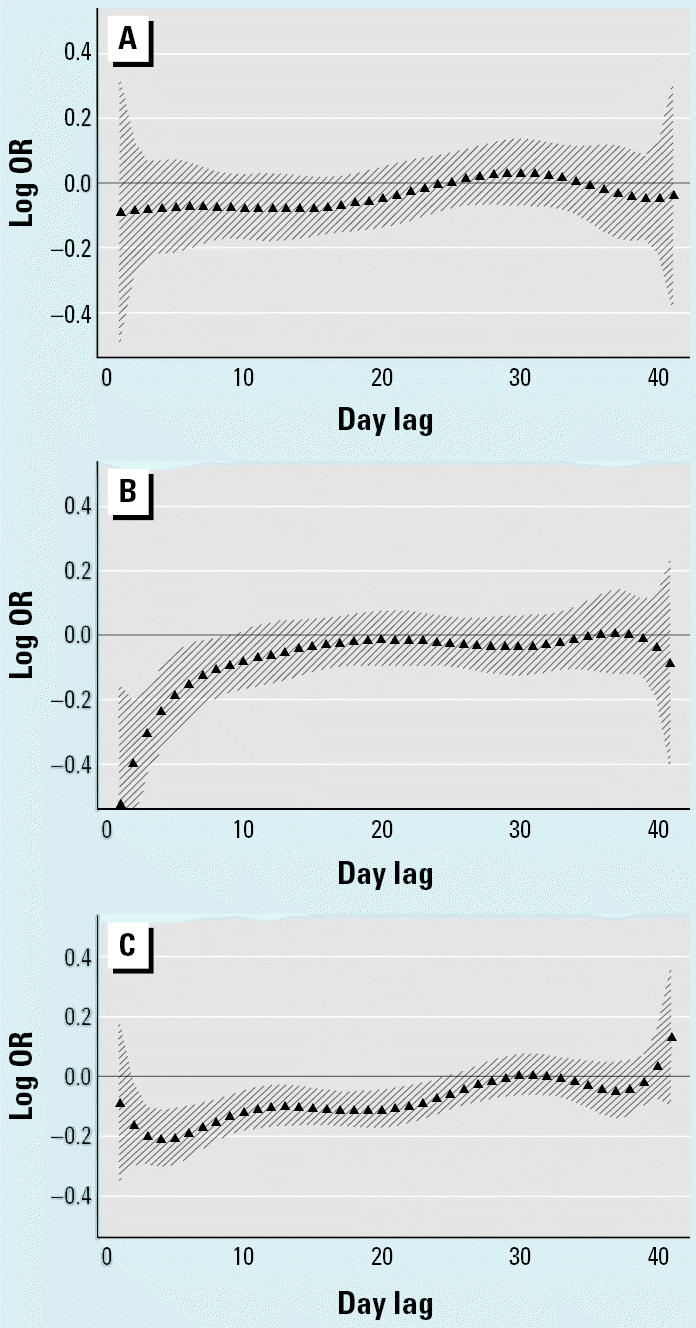
Polynomial distributed lag analysis of total nontrauma mortality versus minimum temperature adjusted for same-day minimum temperature for ages (*A*) 0–64, (*B*) 65–74, and (*C*) ≥75 years. Percent increase in total mortality for each 1°C decrease in minimum daily temperature for lags 1–41 days fitted with a sixth-degree polynomial.

**Figure 3:**
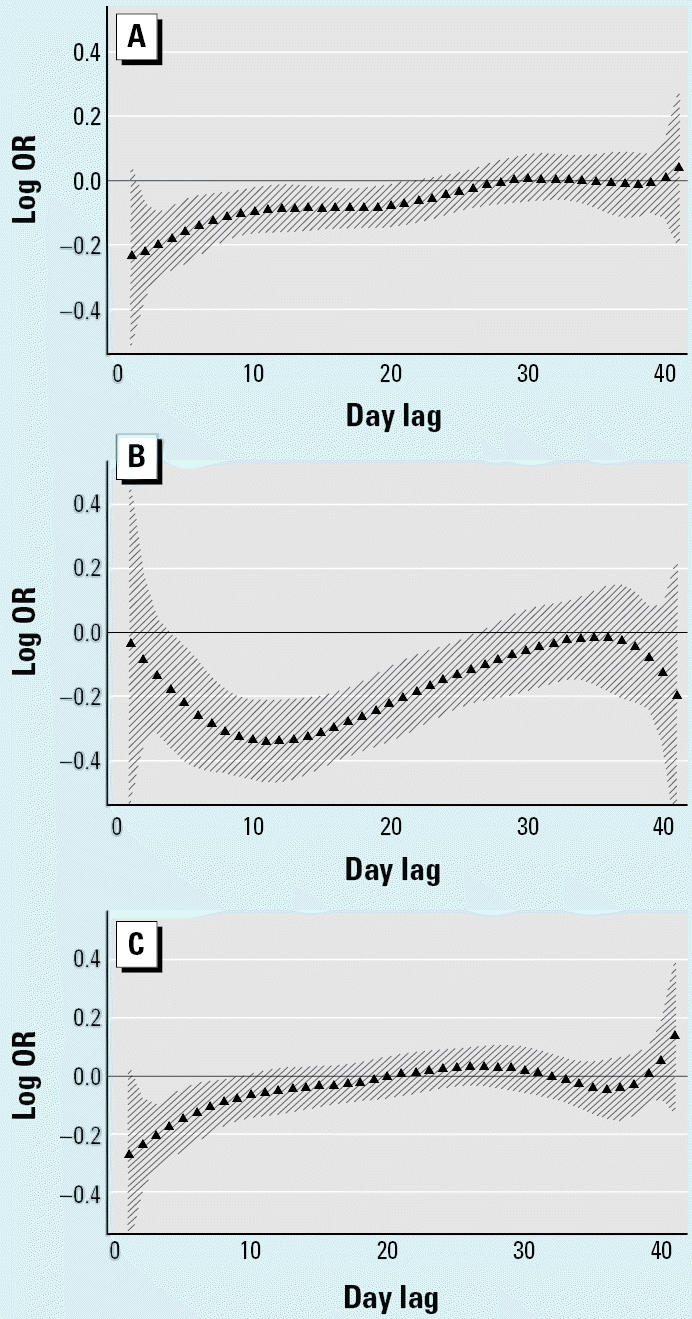
Polynomial distributed lag analysis of (*A*) cardiovascular, (*B*) respiratory, and (*C*) other mortality versus minimum temperature adjusted for same-day minimum temperature. Percent increase in cause-specific mortality for each 1°C decrease in daily minimum temperature for lags 1–41 days fitted with a sixth-degree polynomial.

**Figure 4:**
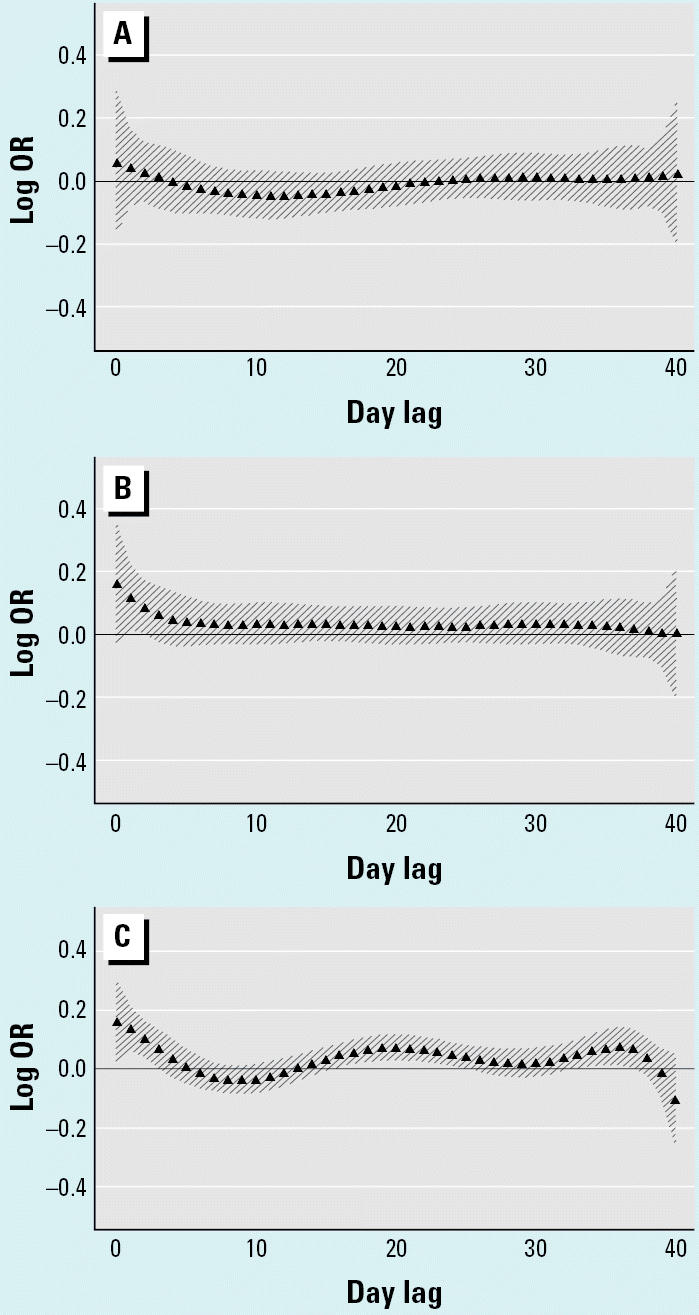
Polynomial distributed lag analysis of total nontrauma mortality versus BS adjusted for minimum temperature for ages (*A*) 0–64, (*B*) 65–74, and (*C*) ≥75 years. Percent increase in total mortality for each 10-μg/m^3^ increase in mean BS for lags 0–40 days fitted with a sixth-degree polynomial.

